# Knowledge, Attitudes, and Practices of School Teachers toward Oral Health in Davangere, India

**DOI:** 10.5005/jp-journals-10005-1413

**Published:** 2017-02-27

**Authors:** Prabhadevi C Maganur, V Satish, Nikhil Marwah, TD Vishwas, MC Dayanand

**Affiliations:** 1 Associate Professor, Department of Pedodontics and Preventive Dental Sciences College of Dentistry, Jazan University, Jazan, Kingdom of Saudi Arabia; 2 Associate Professor, Department of Pedodontics and Preventive Dental Sciences College of Dentistry, Jazan University, Jazan, Kingdom of Saudi Arabia; 3 Head, Department of Pedodontics, Mahatma Gandhi Dental College and Hospital, Jaipur, Rajasthan, India; 4 Professor and Head, Department of Pediatric and Preventive Dentistry, Dr. B.R. Ambedkar Institute of Dental Sciences & Hospital, Patna Bihar, India; 5 Assistant Professor, Department of Oral Surgery, College of Dentistry, Jazan University, Jazan, Kingdom of Saudi Arabia

**Keywords:** Attitude, Knowledge, Practice, Teachers.

## Abstract

**Aim:**

The aim of this study was to assess the knowledge, attitudes, and practices of school teachers toward oral health.

**Settings and design:**

Descriptive study.

**Materials and methods:**

School teachers (n = 150) of Davangere city were recruited into this study. The subjects completed a questionnaire that aimed to evaluate teachers’ knowledge, attitudes, and practices on oral health.

**Statistical analysis:**

The results were statistically analyzed and percentage was calculated.

**Results and conclusion:**

The participants’ oral hygiene habits were found to be regular. The majority of teachers showed good knowledge on oral health. Most of the teachers in this study recognized the importance of oral health. The majority of teachers did incorporate the importance of oral health in teaching and educating children in the school. But, not all teachers are involved effectively. So, the teachers should be trained comprehensively regarding importance of oral health and creating awareness on oral health promotion for their students in combination with health care personnel.

**How to cite this article:**

Maganur PC, Satish V, Marwah N, Vishwas TD, Dayanand MC. Knowledge, Attitudes, and Practices of School Teachers toward Oral Health in Davangere, India. Int J Clin Pediatr Dent 2017;10(1):89-95.

## INTRODUCTION

Education of schoolchildren on oral health is most important because healthy oral habits are developed early in life. The importance of imparting knowledge on oral hygiene to children (infants or preschool or schoolchildren) had been recognized as early as 1878.^1,2^ Children spend considerable amount of time in school, especially during the age when their habits are being formed.^3^ Schools provide an effective platform for promoting oral health because they reach over 1 billion children worldwide.^4^ The school teachers, especially primary school teachers, can play an important role in developing healthy habits in their students.^5,6^ Hence, the role of teachers during these developmental stages of the child is critical.^3^ It is now established that school teachers have an internationally recognized potential role in school-based dental education, and considerable importance has, therefore, been attributed to their dental knowledge.^7^

The use of teachers in health education, however, carries certain disadvantages, the major one being the teacher may be insufficiently trained to deliver such messages; the lack of training on aspects of oral health has shown to prevent teacher from participating in teaching children effectively.^8^ In order to instill a good positive approach to oral habits, the teachers themselves need to have good knowledge, attitudes, and practices toward oral hygiene.

However, previous studies have shown that teachers’ knowledge about oral health was inadequate and was inaccurate in some instances.^8-11^ In contrast, Chikte et al^12^ showed adequate knowledge of teachers on oral health. So, the present survey was done to assess knowledge, attitudes, and practices of oral health in school teachers of Davangere.

## MATERIALS AND METHODS

A total of 150 school teachers were included in this study. In order to assess knowledge, attitudes, and practices regarding oral health, a questionnaire^13^ (Tables 1 to 4) having 31 questions was prepared for school teachers. With the permission from the dean of the school, the dental health questionnaire was explained to the teachers. Later, the questionnaire was distributed to them and collected after 30 minutes.

## RESULTS

The results were tabulated and percentage (100%) was calculated and conclusions were drawn. This survey presented a comprehensive overview of the knowledge, attitudes, and practices of school teachers in Davangere schools.

Table 1 describes knowledge of teachers on oral health. All the teachers in the study knew the fact that oral health does have a role in general health.

Around 36% of teachers concluded that irregular brushing causes decay, 14.7% contributed for gum diseases, 16% of teachers concluded that it will lead to bad breath, 16.7% concluded that irregular brushing causes stains on teeth. Only 17.3% concluded that all the factors are caused due to irregular tooth brushing. About 23.3% of teachers concluded dental problems are due to eating sweets and ice creams. About 58% of teachers agreed for improper brushing. Only 1.3% of teachers concluded that dental problems are due to not visiting the dentist regularly and rinsing mouth. Only 16.7% of teachers pertained to the fact that all the factors cause dental problems. Around 58% of teachers concluded that brushing alone can prevent dental problems, 14% reported that avoiding sticky food and sweets can prevent dental problems and 0.7% teachers concluded that rinsing mouth after meals can prevent dental problems. Only 9.3% of teachers visited the dentist regularly. About 18% of teachers concluded that all the factors are important in prevention of dental problems.

All the teachers (100%) knew that a clean mouth can prevent tooth decay and dentists can clean and polish their teeth. About 56.7% of teachers were aware that their toothpaste contains fluoride and were using it, 12.5% were not using fluoridated toothpaste, and 31.3% were unaware of fluoridated toothpaste. About 46% of teachers were aware of flossing and 54% of teachers were not aware of flossing. Around 47.3% of teachers pertained to the fact that regular cleaning of mouth can prevent bad smell, 14% of teachers accepted that it can reduce the bleeding of gums, 3.3% of teachers concluded that it reduces the loosening of the gums, and 15.3% teachers said that it reduces the loss of teeth. About 20% of teachers accepted that regular cleaning of teeth can reduce all the above-mentioned oral health problems.

When attitudes of teachers on oral health was measured (Table 2), it was observed that all the teachers (100%) accepted the fact that maintenance of oral health is individual responsibility. About 91.3% of teachers accepted that periodical dental visit is required to maintain good oral health and only 7.3% of teachers denied it. Around 81.3% had visited the dentist. Only 18.7% had not visited the dentist. Among these, 31.3% visited with reason of decayed teeth, 20% visited for pain, 8% visited for filling, 3.3% visited for extraction, and 18.7% visited dentist for other reasons.

**Table 1:** **Table 1:** Knowledge of teachers on oral health

K1. Has oral health got any role in general health?					
		*Total*		*Percentage 100%*	
a. Yes		150		100	
b. No		00		00	
c. Do not know		00		00	
K2. What does irregular toothbrushing cause?					
		*Total*		*Percentage 100%*	
a. Decay		54		36	
b. Gum disease		22		14.7	
c. Bad breath		25		16.0	
d. Stains on teeth		24		16.70	
e. Nothing		00		0	
f. All of the above		26		17.3	
K3. Why do we get dental problems?					
		*Total*		*Percentage 100%*	
a. Eating sweets and ice creams		35		23.3	
b. Not brushing properly		87		58	
c. Not rinsing the mouth		02		1.3	
d. Not regularly visiting a dentist		02		1.3	
e. All of the above		25		16.7	
K4. How can you prevent dental problems?					
		*Total*		*Percentage 100%*	
a. Avoiding sweets and sticky foods		21		14	
b. Brushing properly		87		58	
c. Mouthrinsing after meals		01		0.70	
d. Regularly visiting a dentist		14		9.3	
e. All of the above		27		18	
K5. Do you know that a clean mouth can prevent tooth decay?					
		*Total*		*Percentage 100%*	
a. Yes		150		100	
b. No		–		–	
K6. Do you know that a dentist can clean and polish your teeth?					
		*Total*		*Percentage 100%*	
a. Yes		150		100%	
b. No		–		–	
K7. Does your toothpaste contain fluoride?					
		*Total*		*Percentage 100%*	
a. Yes		85		56.7	
b. No		18		12.5	
c. Do not know		47		31.3	
K8. Do you know what floss is?					
		Total		Percentage 100%	
a. Yes		69		46	
b. No		81		54	
K9. Regular cleaning of mouth can prevent					
		*Total*		*Percentage 100%*	
a. Bleeding from gums		21		14	
b. Loosening of gums		05		3.3	
c. Loss of teeth		23		15.3	
d. Bad smell		71		47.3	
e. All of the above		30		20	

**Table 2:** **Table 2:** Attitudes of teachers on oral health

A1. Do you think maintaining a healthy mouth is individual responsibility?					
		*Total*		*Percentage 100%*	
a. Yes		150		100	
b. No		-		–	
A2. Have you visited a dentist before?					
		*Total*		*Percentage 100%*	
a. Yes		122		81.3	
b. No		28		18.7	
A3. If yes, then for what reason?					
		*Total*		*Percentage 100%*	
a. Decay		47		31.3	
b. Pain		30		20	
c. Filling		12		8	
d. Extraction		5		3.3	
e. Any others specify		28		18.7	
A4. Do you think it is required to visit a dentist periodically to maintain the oral health?					
		*Total*		*Percentage 100%*	
a. Yes		139		91.3	
b. No		11		7.3	

**Table 3:** **Table 3:** Practices of teachers on oral health

P1. How do you clean your teeth?					
		*Total*		*Percentage 100%*	
a. Toothbrush and toothpaste		150		100	
b. Toothbrush and toothpowder		–		–	
c. Finger and toothpowder		–		–	
d. Neem sticks		–		–	
e. Any others specify		–		–	
P2. How often you clean your teeth?					
		*Total*		*Percentage 100%*	
a. Once daily		51		34	
b. Twice daily		99		66	
c. More than twice daily		–		–	
d. After every meal		–		–	
P3. How do you brush your teeth?					
		*Total*		*Percentage 100%*	
a. Use horizontal strokes		28		18.70	
b. Use vertical strokes		36		24	
c. Both in horizontal and vertical directions		65		43.3	
d. Circular strokes		21		14	
P4. How often do you change your brush?					
		*Total*		*Percentage 100%*	
a. Once in 3 months		90		60	
b. Once in 6 months		42		28	
c. Yearly once		0		0	
d. When bristles get frayed up		18		12	
e. Do not know exactly		–		–	
P5. What amount of paste do you apply on your brush?					
		*Total*		*Percentage 100%*	
a. Full length of bristles		56		37.3	
b. Half-length of bristles		86		57.4	
c. Pea-sized amount		8		5.3	
P6. Do you press the paste in between the bristles?					
		*Total*		*Percentage 100%*	
a. Yes		62		41.3	
b. No		88		58.7	
P7. Do you rinse your mouth after meals?					
		*Total*		*Percentage 100%*	
a. Yes		93		62	
b. No		13		8.7	
c. Sometimes		44		29.3	
P8. Do you clean your tongue?					
		*Total*		*Percentage 100%*	
a. Yes		150		100	
b. No		00			
P9. How do you clean your tongue?					
		*Total*		*Percentage 100%*	
a. Tongue cleaner		102		68	
b. Fingers		42		28	
c. Toothbrush		6		4	
d. Any others specify		–		–	
P10. Do you know any other oral hygiene aids?					
		*Total*		*Percentage 100%*	
a. Yes		143		95.3	
b. No		7		4.7	
P11. If you know any oral hygiene aids, then, which one do you use?					
		*Total*		*Percentage 100%*	
a. Mouthwash		109		72.6	
b. Dental floss		20		13.3	
c. Tooth picks		21		14	
d. All of the above		00			
e. Any others specify		00			

Table 3 describes practices of teachers in oral health. All the teachers used toothbrush and toothpaste to clean their teeth. Around 34% of teachers brushed their teeth once daily and 66% do brush twice daily. Around 43.3% of teachers did brush their teeth both in the horizontal and vertical directions, 24% teachers did use vertical strokes, 18.7% use horizontal strokes, whereas 14% only used circular strokes while brushing. Majority of teachers (60%) change their toothbrush once in 3 months, 28% changed their toothbrush once in 6 months, and only 12% teachers changed their toothbrush when bristles get frayed up. Around 37.3% of teachers apply the toothpaste to the full length of bristles, 57.4% did apply toothpaste to half-length of the bristles, and only 5.3% used a peanut-sized amount. Only 41.3% of teachers did press the paste in between the bristles, whereas 58.7% did not press paste in between bristles. Nearly 62% teachers did rinse mouth after each meal, 8.7% teachers did not rinse mouth, whereas 29.3% teachers do rinse sometimes. All teachers (100%) did clean their tongue, 68% used tongue cleaner, 28% used finger to clean their tongue, and 4% did use toothbrush to clean their tongue. About 95.3% teachers did know about oral hygiene aids and 4.7% did not know about other oral hygiene aids. Among 95.3% teachers, 72.6% used mouth-wash as oral hygiene aid, 13.3% used dental floss, and only 14% used tooth pick as oral hygiene aid.

Further, (Table 4) general information on topics of oral health in school curriculum was also assessed. All teachers (100%) pertained to the fact that all the above-mentioned topics are present in the school curriculum. Only 83.33% of teachers have been trained to give education on the topics related to the oral health and 16.67% made no attempt to give education on oral health topics. About 6.6% of teachers have given education on teeth types, functions, structure, and eruption to children, 66.67% about brushing, good dietary habits, oral habits, 6.6% have made an attempt to give education on tooth decay gum diseases, irregular teeth, causes, treatment and prevention, and only 3.33% made an attempt to give education on all the above-mentioned topics. Around 20% teachers have used oral health talks as a method of teaching schoolchildren about oral hygiene. About 56.67% used models, chart, and poster as mode of teaching. Only 6.67% used all methods to educate children on oral health.

Almost all their schoolchildren (100%) have responded favorably to the teachers for oral health education, and all the schoolchildren are benefited by oral health education given by the teachers who have been trained.

## DISCUSSION

Preprimary and primary schools have a great potential for influencing the health behavior of the child.^14-17^ During this period, the child goes through active developmental stages. The role of teachers during these developmental stages of the child is very important. Hence, school teachers can play a major role in oral health education programs at school levels. Schools have tremendous capacity to be supportive of programs involving preventive dentistry for children.^18^ It was found that teachers traditionally have educated children regarding oral health and often participated in school-based prevention programs.^19^

**Table 4:** **Table 4:** General information on oral health in school curriculum

G1. Are the topics related to teeth and mouth in the present school curriculum?					
		*Total*		*Percentage 100%*	
a. Yes		150		100	
b. No		00		00	
G2. Have you been trained to give education on topics related to teeth and mouth to schoolchildren?					
		*Total*		*Percentage 100%*	
a. Yes		125		83.33	
b. No		25		16.67	
G3. Have you made an attempt to give education related to teeth and mouth to your students?					
		*Total*		*Percentage 100%*	
a. Yes		125		83.33	
b. No		25		16.67	
G4. If yes, what kind of oral health education have you given to your schoolchildren?					
		*Total*		*Percentage 100%*	
a. About the teeth types, function structure, and eruption		10		6.6	
b. About brushing, good dietary habits, injurious oral habits		100		66.67	
c. Education about tooth decay, gum diseases, irregular teeth, their causes, treatment, and prevention		10		6.6	
d. All of the above		5		3.33	
G5. What methods are you employing to give oral education to schoolchildren?					
		*Total*		*Percentage 100%*	
a. Oral health talks		30		20	
b. Models, charts, and posters		85		56.67	
c. All of the above		10		6.67	
G6. How have your students responded to oral health education?					
		*Total*		*Percentage 100%*	
a. Favorably		125		100	
b. Unfavorably		–		–	
G7. Do you think oral health education has benefited your schoolchildren?					
		*Total*		*Percentage 100%*	
a. Yes		125		100	
b. No		00		00	

Our aim in this study was to assess the oral health knowledge and attitudes of school teachers toward oral health practice by administering self-administered questionnaires. The use of self-administered questionnaires has its own limitations. We have tried to overcome the bias by recording the data confidentially and explaining to the subjects the importance of the survey before filling the questionnaire. Moreover, this method of collecting data has been previously tested and has shown adequate reliability.^6,7,9,15^

Around 36% of teachers in the present study were aware of decay and 14.7% attributed that irregular brushing causes gum diseases. Teachers of our study showed more knowledge on effects of irregular brushing when compared with the studies done by Khan et al^20^ and al-Tamimi and Petersen,^5^ who showed that 26% of school teachers did not know anything with regard to tooth decay and one-third did not know anything about gum disease.

In the present survey, 23.3% of teachers ascertained eating sweets and ice creams causes tooth decay. This conclusion is on par with studies conducted by Petersen et al.^15^ About 14% teachers attributed that avoiding sticky foods decreases dental problems, which is similar with the study conducted by Ramroop et al^21^ in which two-thirds of teachers thought dental decay could be prevented by eating less sugar.

Fluoridated containing compounds have been used in preventing incipient carious lesion since the early 1900s.^22^ Optimal water fluoridation has been recognized as the single most cost effective public health measure known to science for preventing tooth decay.^7^ Hence it was deemed essential to determine the knowledge and attitudes of schoolteachers towards the subjects of fluorides. In our study, more than 50 % of the teachers were aware of fluoridated tooth pastes which was similar to the study conducted on South African teachers^12^ who showed adequate knowledge on fluorides. But in contrast to this, around 37.3% were unaware of fluoridated tooth paste in our study. This was similar to study done by Ankita Mota et al^3^ and HD Sgan et al^7^ who reported half of the teachers were never aware of fluoridated tooth paste.

About 54% were unaware of floss. These results indicate that improvement in knowledge toward the use of dental floss is needed, as floss helps to remove plaque and other debris interdentally. Intervention to increase the knowledge and subsequent use of flossing is essential and is in agreement with other studies.^23^ Similar opinion was seen in the study conducted by Mota et al^3^ where percentage of teachers using floss was very low.

According to our study, the knowledge on oral health among school teachers was quiet impressive.

### Attitudes

All the teachers were aware of the importance of a healthy mouth. Most of the teachers visited the dentist if there was a problem. This view was very similar to the study conducted by Almas et al^24^ and Paul Lang et al.^9^ However, the teachers know the importance of visiting a dentist regularly. This was in concurrence with the study conducted by Ramroop et al^21^ and Chikte et al.^12^

### Practices

Knowledge and practice in brushing teeth, changing toothbrush, and amount of toothpaste to be used were quite satisfactory.

All the teachers used toothbrush and toothpaste to clean their teeth. Around 34% of teachers brushed their teeth once daily, 66% do brush twice daily. The results are similar to the study conducted by Chikte et al^12^ and Al-Mansour and Al-Zarea.^25^

In our study, the teachers exhibited knowledge on brushing techniques. This was on par with the study conducted by Almas et al,^24^ who also said the teachers showed high level of knowledge regarding brushing.

About 72.6% used mouthwash as an oral hygiene aid, 13.3% used dental floss, and only 14% used toothpicks as oral hygiene aids. Interestingly, many of the teachers did use mouthwash as an accessory tooth cleaning aid. This was on par with a study by Mota et al.^3^ While the reason behind this discrepancy could not be ascertained, one possible explanation could be the active promotion of mouthwashes in visual media and newspapers.^3^

### General

In our survey, all teachers (100%) pertained to the fact that topics on oral health were included in the present school curriculum. The majority of school teachers have been trained to give education on the topics related to the oral health, and they made an attempt to give education on the oral health topics to their children in the school. Because of insufficient knowledge, there were few teachers who were not presenting lectures to the children on oral health. All of the teachers, irrespective of their experience, had highly acceptable scores for attitudes toward oral health. This showed that although some did not have good knowledge, they still had positive attitudes regarding their oral health. The results were similar to the previous study conducted by Wyne et al,^26^ Ahmad,^17^ and Sukhabogi et al.^27^

Numerous studies have been conducted worldwide and demonstrated the attitude, knowledge, practices, and willingness of school teachers to promote oral health among their children in school. Some studies in Minnesota, USA,^11^ Michigan USA,^9^ and in some parts of India^3,16,27^ have shown inadequate oral health knowledge, while on the contrary, studies from Romania, China,

Saudi Arabia, and United States of America have reported positive attitudes and knowledge on oral health among teachers, and that they showed willingness to participate in oral health promotion.^5,6,15,17^ Hence, providing education on oral health in schools helps children to develop personal skills, provides knowledge about oral health, and promotes positive attitudes and healthy behaviors. Oral health education can be taught as a specific subject or as part of other subjects, addressing the underlying physical, psychological, cultural, and social determinants of oral and general health.^4^

The results of such questionnaire surveys should always be viewed with caution. There is possibility of bias created. This is created especially

- When the respondents are aware that the survey is being conducted by dental specialists;
- When filling favorite responses to the questions in the questionnaire.

So, we conclude the following:

- Even though most of the teachers show satisfactory knowledge in some aspects of preventive oral health, they still lag behind in knowledge in some crucial parts of oral health. There is a definite and immediate need for teacher training programs on basic oral health knowledge. Further workshops are recommended to improve their existing knowledge.
- All the teachers should be trained at regular intervals, comprehensively regarding importance of oral health and creating awareness on oral health promotion for their students with the help of health care personnel or organizations.
- Presently, the school curriculum has topics on oral health and its importance. Teachers can be considered to educate and motivate schoolchildren in maintaining their oral health. Efforts should be made to involve all teachers to educate and teach the child.
